# Hospitalization and Survival of Medicare Patients Treated With Carboplatin Plus Paclitaxel or Pemetrexed for Metastatic, Nonsquamous, Non–Small Cell Lung Cancer

**DOI:** 10.1001/jamanetworkopen.2018.3023

**Published:** 2018-10-05

**Authors:** Gabriel A. Brooks, Andrea M. Austin, Hajime Uno, Konstantin H. Dragnev, Anna N. A. Tosteson, Deborah Schrag

**Affiliations:** 1 The Dartmouth Institute for Health Policy and Clinical Practice, Geisel School of Medicine, Lebanon, New Hampshire; 2 Department of Medicine, Geisel School of Medicine, Lebanon, New Hampshire; 3 Norris Cotton Cancer Center, Lebanon, New Hampshire; 4 Division of Population Sciences, Dana-Farber Cancer Institute, Boston, Massachusetts

## Abstract

**Importance:**

Chemotherapy is a mainstay treatment of metastatic non–small cell lung cancer. However, little is known about the comparative risk of hospitalization associated with commonly used chemotherapy regimens.

**Objective:**

To evaluate the real-world association of specific lung cancer chemotherapy regimens with measures of acute hospital care (primary objective) and survival (secondary objective).

**Design, Setting, and Participants:**

Retrospective, propensity-matched, cohort study using the Surveillance, Epidemiology, and End Results–Medicare linked data for cancer diagnoses between 2008 and 2013, with follow-up through 2014. Patients were Medicare beneficiaries 66 years of age or older receiving initial chemotherapy for treatment of stage IV, nonsquamous, non–small cell lung cancer. Analyses were performed between September 2017 and April 2018.

**Exposures:**

Receipt of chemotherapy with carboplatin-pemetrexed or carboplatin-paclitaxel, with or without concurrent bevacizumab.

**Main Outcomes and Measures:**

The primary outcome was risk of hospitalization within 30 days of chemotherapy initiation. Secondary measures included cumulative 90-day hospitalizations, 90-day mean hospital-free survival time, and overall survival.

**Results:**

Of the 3310 eligible patients, 1823 received carboplatin-pemetrexed, and 1487 received carboplatin-paclitaxel. The median age at diagnosis was 73 years (interquartile range, 69-77 years), 1784 patients (53.9%) were men, and 2909 patients (87.9%) were non-Hispanic white. In total, 2182 patients were included in the propensity-matched analysis. The 30-day hospitalization risk was 20.7% (95% CI, 18.3%-23.1%) among patients receiving carboplatin-pemetrexed vs 26.0% (95% CI, 23.4%-28.6%) among patients receiving carboplatin-paclitaxel (5.3% difference; *P* = .003). The 90-day cumulative hospitalizations did not differ significantly between the 2 treatment groups; however, the 90-day mean hospital-free survival time was improved for patients receiving carboplatin-pemetrexed (68.4 days [95% CI, 66.5-70.4 days] vs 63.6 days [95% CI, 61.6-65.7 days]; *P* = .001). The median survival time was 9.0 months (95% CI, 8.4-9.5 months) with carboplatin-pemetrexed therapy vs 7.6 months (95% CI, 7.0-8.4 months) with carboplatin-paclitaxel therapy (*P* = .005). Stratified analyses showed no association of bevacizumab use with hospitalization risk or survival when combined with either chemotherapy regimen.

**Conclusions and Relevance:**

The findings from this study suggest that patients receiving carboplatin-pemetrexed compared with those receiving carboplatin-paclitaxel have a lower 30-day hospitalization risk, a greater 90-day hospital-free survival, and improved overall survival. Information about hospitalization risk may provide valuable context for evaluating real-world cancer treatment outcomes.

## Key Points

**Question:**

Does the risk of hospitalization among elderly patients receiving chemotherapy for metastatic, nonsquamous, non–small cell lung cancer vary by chemotherapy regimen?

**Findings:**

In a cohort study of 2182 propensity-matched Medicare beneficiaries with non–small cell lung cancer, the 30-day hospitalization risk was 5% lower for patients receiving carboplatin with pemetrexed (21%) than for those receiving carboplatin with paclitaxel (26%), a statistically significant difference.

**Meaning:**

When multiple standard-of-care chemotherapy regimens are available in a specific treatment setting, measures of regimen-specific hospitalization risk may be relevant for treatment selection.

## Teaser

This cohort study analyzes Medicare claims linked with Surveillance, Epidemiology, and End Results (SEER) program data to assess hospitalization risk and survival among US patients receiving carboplatin-paclitaxel or carboplatin-pemetrexed for treatment of metastatic, nonsquamous, non–small cell lung cancer.

## Introduction

The outcomes of palliative chemotherapy for cancer are commonly assessed in terms of overall survival. The use of survival as a summary outcome measure is necessary but not sufficient for evaluating and describing the effectiveness of palliative-intent cancer therapy because patients value not only survival but also quality of life and avoiding adverse effects. Because clinical trials are typically conducted in populations that are younger and healthier than those encountered in practice, observational data sources—including medical records, tumor registries, administrative claims, and, in particular, the Surveillance, Epidemiology, and End Results (SEER)–Medicare linked database—are an important source of information about the relative benefits and harms of alternative chemotherapy regimens.

However, claims-based measures of drug toxicity and adverse events are underdeveloped. “Hospitalization indicated” is a general criterion for classifying chemotherapy-related adverse events as grade 3 (severe) using the Common Terminology Criteria for Adverse Events of the National Cancer Institute,^1^ and there is growing acknowledgment that hospitalization itself can be an adverse effect of cancer treatment. Hospitalization of patients with cancer is common,^2,3^ variable across regions and practices,^4,5^ and potentially avoidable.^6^ Furthermore, prior studies have shown that chemotherapy regimen selection can be associated with hospitalization risk.^7,8^

The present study compares the hospitalization and survival rates among Medicare beneficiaries receiving the 2 most common cytotoxic chemotherapy regimens for metastatic, nonsquamous, non–small cell lung cancer: carboplatin with paclitaxel or carboplatin with pemetrexed. This analysis evaluates whether the choice of chemotherapy regimen was associated with hospitalization as captured by 3 alternative metrics. We secondarily evaluated survival among these patients.

## Methods

### Study Design and Data Source

We performed a retrospective cohort study of Medicare beneficiaries receiving first-line chemotherapy for metastatic, nonsquamous, non–small cell lung cancer with either carboplatin-paclitaxel or carboplatin-pemetrexed (either regimen with or without bevacizumab). The data source for this study was the SEER-Medicare linked data for patients who received a diagnosis of lung cancer between 2008 and 2013 (representing the most current release of the SEER-Medicare linked data at the time of publication, with survival data through December 31, 2014). The SEER program of the National Cancer Institute collects uniformly reported data from population-based cancer registries, including cancer site, stage, diagnosis date, and other clinical variables.^9^ The SEER program data cover approximately 28% of the US population. Since 1991, the National Cancer Institute has linked SEER data with Medicare administrative data for more than 94% of SEER registry patients 65 years of age or older.^10^ The linked Medicare data used in this analysis included deidentified fee-for-service claims and event records from inpatient, outpatient, carrier, durable medical equipment, and Part D event files. The research question, study design, and research outcomes (primary and secondary) were all prespecified in the research protocol, as recommended in the International Society for Pharmacoeconomics and Outcomes Research (ISPOR) task force report on good research practices.^11^ The study was approved by the Dartmouth College institutional review board, which also waived the need for obtaining informed patient consent because the data were deidentified, and was performed under a data use agreement with the National Cancer Institute.

### Patients

We identified all patients in the SEER-Medicare linked data who had received a new diagnosis of metastatic non–small cell lung cancer between 2008 and 2013 (stage IV as classified by the American Joint Committee on Cancer’s *AJCC Cancer Staging Manual*, 6th edition). We subsequently excluded patients with a squamous histologic subtype to focus our analysis on patients who were candidates to receive the chemotherapy regimens of interest. We further restricted our sample to patients who were 66 years of age or older at the time of diagnosis and who had continuous enrollment in fee-for-service Medicare (Parts A and B) in the 12 months before and 6 months after cancer diagnosis (or until death) to ensure adequate measurement of baseline comorbidities and posttreatment outcomes. Additional exclusions included prior cancer diagnosis (other than localized breast, colon, or prostate cancer) or concurrent radiation therapy (defined as receipt of radiation therapy on the same day as chemotherapy initiation). The final analytic cohort was restricted to patients receiving first-line treatment with either carboplatin-pemetrexed or carboplatin-paclitaxel (with or without concurrent bevacizumab).

### Exposure and Outcome Definitions

The main exposure of interest was the initial chemotherapy regimen. We classified regimens by evaluating the first date of receipt of any chemotherapy agent (index date) as captured in Medicare claims from the outpatient, carrier, durable medical equipment, or Part D event files within 90 days of the first day of the diagnosis month. We then identified all chemotherapy claims from within 7 days of the index date and assigned the chemotherapy regimen based on all chemotherapy agents received within this period.

The prespecified primary outcome measure was 30-day hospitalization risk, defined as the risk of acute inpatient hospital admission within 30 days of the first chemotherapy treatment. Additional hospitalization measures were 90-day cumulative hospitalizations (the count of acute inpatient hospitalizations) and 90-day mean hospital-free survival time (the mean number of days to the first occurrence of either hospitalization or death, restricted to a maximum value of 90 days). The restricted mean survival time has been endorsed as a nonparametric (model-free) measure that is relatively intuitive^12^; incorporating hospitalizations into the restricted mean survival time provides a framework for evaluating both death and the semicompeting risk of hospitalization within a single measure. We assessed overall survival at 90 days and at the median survival time.

### Statistical Analysis

We performed descriptive analyses of patient clinical and sociodemographic characteristics, comparing the 2 patient cohorts (patients receiving carboplatin-pemetrexed vs carboplatin-paclitaxel) using 2-sided *t* tests for continuous variables and χ^2^ tests for categorical variables. Given observed imbalances in some of these characteristics, we then used propensity score matching to balance the observable baseline characteristics across the cohorts.

The propensity model evaluated the probability that a patient would receive carboplatin-pemetrexed (vs carboplatin-paclitaxel). Demographic and clinicopathologic variables included in the propensity model were age group, sex, race/ethnicity, marital status, year of cancer diagnosis, Charlson Comorbidity Index (without points for cancer diagnosis, categorized as 0, 1, or ≥2; higher scores indicating a greater number of noncancer comorbid conditions),^13,14^ *International Classification of Diseases for Oncology, Third Revision* (*ICD-O-3*) site and histologic codes, receipt of radiation treatment within the prior 30 days, and hospitalization within the prior 180 days. Additional variables included in the propensity model were area-level measures taken from the SEER data, including measures of census tract poverty, urban-rural residence classification, and US geographic region of residence as well as a claims-based indicator for chemotherapy treatment at hospital-based vs office-based practice sites. Further details of the propensity model are presented in Table 1. We then used a nearest-neighbor matching algorithm without replacement to obtain a 1:1 match; matches were constrained within an optimal caliper equal to 0.2 times the SD of the logit of the propensity scores.^15,16,17^ The postmatch covariate balance was verified by calculating the standardized difference of mean values for each factor in the 2 groups.

**Table 1.** Characteristics of Patients With Stage IV Lung Cancer Treated With Carboplatin With Paclitaxel or Carboplatin With Pemetrexed

Characteristic^a^	All Patients			Propensity-Matched Patients		
	No. (%)		*P* Value	No. (%)		Standardized Difference
	Carboplatin-Paclitaxel	Carboplatin-Pemetrexed		Carboplatin-Paclitaxel	Carboplatin-Pemetrexed	
No.	1487	1823		1091	1091	
Age group, y						
66-69	410 (27.6)	476 (26.1)	.19	296 (27.1)	298 (27.3)	−0.051
70-74	506 (34.0)	614 (33.7)		378 (34.6)	381 (34.9)	−0.021
75-79	370 (24.9)	438 (24.0)		267 (24.5)	267 (24.5)	0.019
≥80	201 (13.5)	295 (16.2)		150 (13.7)	145 (13.3)	0.060
Sex						
Male	805 (54.1)	979 (53.7)	.80	592 (54.3)	600 (55.0)	0.005
Female	682 (45.9)	844 (46.3)		499 (45.7)	491 (45.0)	−0.005
Race/ethnicity						
White, non-Hispanic	1304 (87.7)	1604 (88.0)	.80	956 (87.6)	960 (88.0)	0.042
Other	183 (12.3)	219 (12.0)		135 (12.4)	131 (12.0)	−0.042
Marital status						
Married/domestic partner	895 (60.2)	1143 (62.7)	.14	662 (60.7)	659 (60.4)	0.032
Unmarried	592 (39.8)	680 (37.3)		429 (39.3)	432 (39.6)	−0.032
Diagnosis year						
2008	329 (22.1)	48 (2.6)	<.001	48 (4.4)	48 (4.4)	0.000
2009	271 (18.2)	210 (11.5)		179 (16.4)	204 (18.7)	−0.053
2010	237 (15.9)	316 (17.3)		223 (20.4)	253 (23.2)	−0.110
2011	214 (14.4)	369 (20.2)		210 (19.2)	194 (17.8)	0.002
2012	221 (14.9)	434 (23.8)		219 (20.1)	196 (18.0)	0.064
2013	215 (14.5)	446 (24.5)		212 (19.4)	196 (18.0)	0.032
Charlson Comorbidity Index^b^						
0	690 (46.4)	810 (44.4)	.53	503 (46.1)	499 (45.7)	−0.064
1	424 (28.5)	539 (29.6)		317 (29.1)	316 (29.0)	0.052
≥2	373 (25.1)	474 (26.0)		271 (24.8)	276 (25.3)	0.018
*ICD-O-3* site code						
Upper lobe of lung	52 (3.5)	51 (2.8)	.58	34 (3.1)	40 (3.7)	−0.025
Middle or lower lobe of lung	719 (48.4)	905 (49.6)		542 (49.7)	543 (49.8)	−0.025
Bronchus	438 (29.5)	543 (29.8)		318 (29.1)	303 (27.8)	0.052
Other or overlapping sites	278 (18.7)	324 (17.8)		197 (18.1)	205 (18.8)	−0.021
Histology						
Adenocarcinoma	1429 (96.1)	1796 (98.5)	<.001	1069 (98)	1064 (97.5)	0.005
Large cell	58 (3.9)	27 (1.5)		22 (2.0)	27 (2.5)	−0.005
Prior radiation therapy^c^						
Yes	1176 (79.1)	1391 (76.3)	.06	857 (78.6)	862 (79.0)	0.046
No	311 (20.9)	432 (23.7)		234 (21.4)	229 (21.0)	−0.046
Recent hospitalizations^d^						
None	745 (50.1)	872 (47.8)	.40	547 (50.1)	560 (51.3)	−0.081
1	548 (36.9)	694 (38.1)		400 (36.7)	398 (36.5)	0.049
≥2	194 (13.0)	257 (14.1)		144 (13.2)	133 (12.2)	0.045
Census tract poverty rate						
0 to <5% poverty	315 (21.2)	476 (26.1)	<.001	240 (22)	216 (19.8)	0.135
5% to <10% poverty	376 (25.3)	423 (23.2)		269 (24.7)	283 (25.9)	−0.079
10% to <20% poverty	411 (27.6)	415 (22.8)		284 (26)	306 (28.0)	−0.063
≥20% poverty	258 (17.4)	228 (12.5)		174 (15.9)	188 (17.2)	−0.045
Unknown	127 (8.5)	281 (15.4)		124 (11.4)	98 (9.0)	0.013
Urban-rural classification						
Large metropolitan region	746 (50.2)	1096 (60.1)	<.001	581 (53.3)	517 (47.4)	0.074
Metropolitan region	455 (30.6)	457 (25.1)		313 (28.7)	346 (31.7)	−0.025
Urban	100 (6.7)	103 (5.7)		70 (6.4)	81 (7.4)	−0.033
Less urban/rural	185 (12.4)	167 (9.2)		127 (11.6)	147 (13.5)	−0.077
Region						
Northeast	212 (14.3)	237 (13.0)	<.001	163 (14.9)	175 (16.0)	0.188
Southeast	288 (19.4)	485 (26.6)		220 (20.2)	177 (16.2)	−0.145
Midwest	447 (30.1)	408 (22.4)		301 (27.6)	349 (32.0)	−0.092
West	540 (36.3)	693 (38.0)		407 (37.3)	390 (35.7)	−0.021
Treatment setting^e^						
Hospital outpatient	995 (66.9)	1040 (57.0)	<.001	690 (63.2)	700 (64.2)	0.074
Office-based	492 (33.1)	783 (43.0)		401 (36.8)	391 (35.8)	−0.074
Bevacizumab use, cycle 1^f^						
Yes	499 (33.6)	414 (22.7)	<.001	361 (33.1)	244 (22.4)	NA

Abbreviations: *ICD-O-3*, *International Classification of Diseases for Oncology, Third Revision*; NA, not applicable.

^a^ Except as specified, all listed characteristics are included in the propensity model.

^b^ Per Charlson-Deyo-Klabunde method.^13,14^ Higher scores indicate a greater number of noncancer comorbid conditions.

^c^ In 30 days prior to chemotherapy initiation.

^d^ In 180 days prior to chemotherapy initiation.

^e^ Derived from the Medicare claims file in which the index chemotherapy claim was found. Claims from hospital-based practices appear in the outpatient file.

^f^ Characteristic not included in the propensity model; *P* < . 001 for difference in bevacizumab use in propensity-matched patients (determined by use of the χ^2^ test).

After propensity score matching, we assessed the study outcomes of 30-day hospitalization risk (primary outcome), 90-day cumulative hospitalizations, and 90-day mean hospital-free survival time. For overall survival, we report both the 90-day overall survival probability and the median overall survival time (as calculated by the Kaplan-Meier method). Main outcome measures are reported with 95% CIs and *P* values. We also report descriptive characteristics of hospitalizations occurring within 90 days of chemotherapy initiation stratified by chemotherapy regimen, including information about common admission diagnoses and hospital length of stay. We assessed for differences in the mean length of stay with a 2-sample Wilcoxon test. Admission diagnoses were identified using *International Classification of Diseases, Ninth Revision, Clinical Modification* codes on inpatient hospital claims from the inpatient file, and diagnosis codes were grouped into descriptive categories by one of us (G.A.B).

Finally, we performed additional analyses to evaluate the association of bevacizumab with 30-day hospitalization risk and overall survival. These stratified analyses were performed within the prematch patient cohort and are reported separately for each of the studied chemotherapy regimens. For the analysis of 30-day hospitalization risk, we fitted multivariable logistic regression models in which the main exposure of interest was receipt of bevacizumab. For the analysis of overall survival, we used Cox proportional hazards models to evaluate the association of bevacizumab use with mortality. Each of these analyses was adjusted for the same covariates included in the previously described propensity model.

A 2-sided *P* ≤ .05 was considered statistically significant. Analyses were conducted between September 2017 and April 2018 using Stata, version 15 (StataCorp); R, version 3.1.1 (packages survRM2^18^ and surv2sampleComp^19^); and SAS, version 9.4 (SAS Institute Inc).

## Results

### Patients

**Figure 1.**
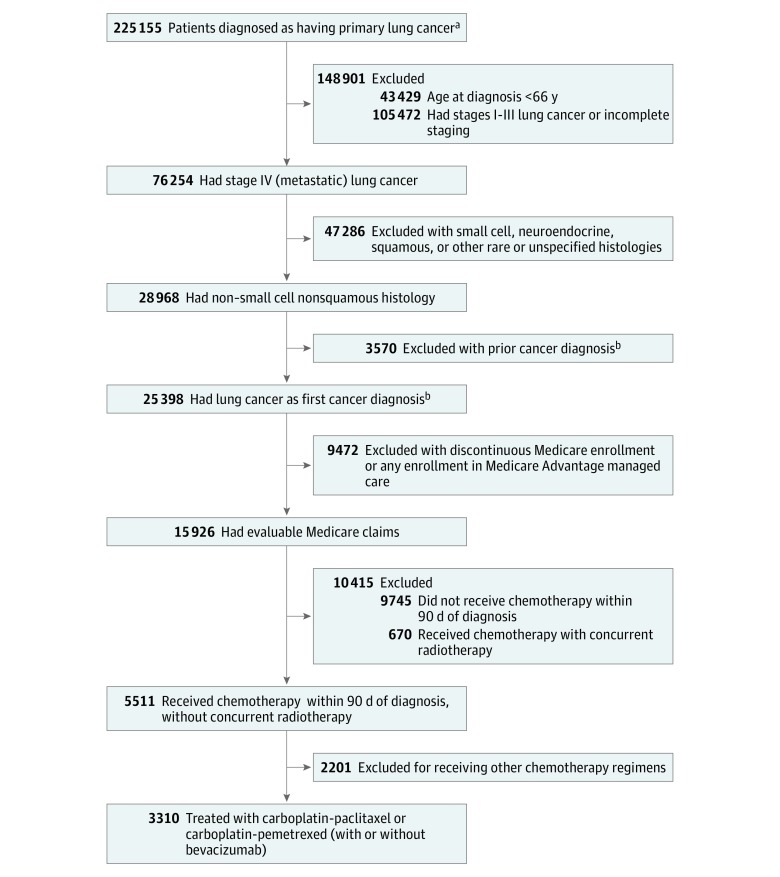
Cohort Selection for Patients Receiving Chemotherapy for Stage IV, Nonsquamous, Non–Small Cell Lung Cancer ^a^Lung cancer diagnosis between 2008 and 2013 not from autopsy or death certificate and without missing diagnosis month for patients without end-stage renal disease. ^b^Prior diagnosis with localized breast, colon, or prostate cancer was permitted.

In total, 3310 patients with stage IV, nonsquamous, non–small cell lung cancer met study inclusion criteria, including 1487 patients receiving carboplatin-paclitaxel and 1823 patients receiving carboplatin-pemetrexed. Details of the cohort selection are shown in Figure 1. The median age at diagnosis was 73 years (interquartile range, 69-77 years), 1784 patients (53.9%) were men, and 2909 patients (87.9%) were non-Hispanic white.

Prior to propensity score matching, patients receiving carboplatin-pemetrexed and patients receiving carboplatin-paclitaxel were similar by age, sex, race/ethnicity, marital status, Charlson Comorbidity Index, and *ICD-O-3* site code (Table 1). However, there were significant differences by diagnosis year (with increasing use of carboplatin-pemetrexed in more recent years) and histology (adenocarcinoma vs large cell carcinoma). Propensity score matching yielded 2182 matched patients (1091 receiving each regimen, a 73% match rate), and matching achieved satisfactory balance across variables included in the propensity model (with absolute standardized differences of less than 0.2 for all strata of variables in the propensity match) (Table 1). Bevacizumab use was more common among patients receiving carboplatin-paclitaxel than among those receiving carboplatin-pemetrexed, both before and after propensity matching (361 [33.1%] vs 244 [22.4%] among propensity-matched patients [*P* < .001]). Use of bevacizumab was considered to be nonindependent from the choice of chemotherapy regimen (the US Food and Drug Administration approval is specific to use with carboplatin-paclitaxel), and bevacizumab use was not included in the propensity model.

### Hospitalization and Survival by Regimen

The 30-day hospitalization risk was 20.7% (95% CI, 18.3%-23.1%) among patients receiving carboplatin-pemetrexed and 26.0% (95% CI, 23.4%-28.6%) among patients receiving carboplatin-paclitaxel, corresponding to a 5.3% lower risk of hospitalization with carboplatin-pemetrexed (*P* = .003). There were also fewer cumulative hospitalizations at 90 days among patients receiving carboplatin-pemetrexed vs carboplatin-paclitaxel (585 vs 647 hospitalizations); however, the difference was not statistically significant (*P* = .11). The 90-day mean hospital-free survival time was significantly longer among patients receiving carboplatin-pemetrexed than among patients receiving carboplatin-paclitaxel (68.4 days [95% CI, 66.5-70.4 days] vs 63.6 days [95% CI, 61.6-65.7 days]; *P* = .001). Findings are summarized in Table 2.

**Table 2.** Hospitalization and Survival Outcomes in Propensity-Matched Patients Receiving Carboplatin-Based Chemotherapy With Paclitaxel or Pemetrexed

Outcome	Carboplatin-Paclitaxel (n = 1091)	Carboplatin-Pemetrexed (n = 1091)	Difference	*P* Value
30 Days				
Hospitalization risk, % (95% CI)	26.0 (23.4 to 28.6)	20.7 (18.3 to 23.1)	−5.3 (−1.8 to −8.9)	.003
90 Days				
Hospitalization count, mean (95% CI), No.	0.59 (0.54 to 0.64)	0.54 (0.49 to 0.59)	−0.05 (−0.01 to −0.12)	.11
Hospital-free survival time, mean (95% CI), d	63.6 (61.6 to 65.7)	68.4 (66.5 to 70.4)	4.8 (2.0 to 7.6)	.001
Overall survival probability, % (95% CI)	75.3 (72.6 to 77.8)	82.1 (79.9 to 84.4)	6.8 (3.5 to 10.3)	<.001
Overall survival, median (95% CI), mo	7.6 (7.0 to 8.4)	9.0 (8.4 to 9.5)	1.4 (0.4 to 2.4)	.005

The mean hospital length of stay did not differ between patients receiving carboplatin-pemetrexed and patients receiving carboplatin-paclitaxel (5.7 vs 5.5 days; *P* = .13). The most common reasons for admission were respiratory tract complications, fever or infection, and cancer (lung cancer or metastasis); admission diagnosis codes from these categories accounted for 40% of all hospitalizations. Category distributions of admission diagnosis codes were similar for both chemotherapy regimens, including similar numbers of hospitalizations for fever or infection (100 hospitalizations of patients receiving carboplatin-paclitaxel vs 73 hospitalizations of patients receiving carboplatin-pemetrexed) and for neutropenia (15 vs 11 hospitalizations).

**Figure 2.**
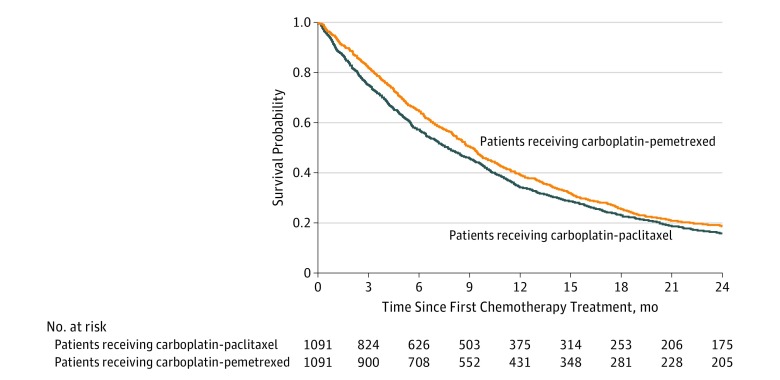
Overall Survival Among Propensity-Matched Patients Receiving Carboplatin-Based Chemotherapy With Pemetrexed or Paclitaxel

Both the 90-day survival probability and the median overall survival were modestly but significantly improved for patients receiving carboplatin-pemetrexed compared with patients receiving carboplatin-paclitaxel (Table 2). The median survival was 9.0 months (95% CI, 8.4-9.5 months) with carboplatin-pemetrexed vs 7.6 months (95% CI, 7.0-8.4 months) with carboplatin-paclitaxel (*P* = .005); Figure 2 shows Kaplan-Meier curves of overall survival. Hospitalization and survival outcomes from the prematch (crude) cohorts were consistent with the findings from the main propensity-matched analysis (eTable in Supplement 1).

### Association of Bevacizumab With Hospitalization and Survival

There was no evidence of an association between bevacizumab use and hospitalization among patients receiving either carboplatin-pemetrexed (adjusted odds ratio, 0.88; 95% CI, 0.66-1.18) or carboplatin-paclitaxel (adjusted odds ratio, 0.96; 95% CI, 0.76-1.28). In addition, bevacizumab use was not associated with differences in overall survival among patients receiving either carboplatin-pemetrexed (adjusted hazard ratio, 1.04; 95% CI, 0.93-1.17) or carboplatin-paclitaxel (adjusted hazard ratio, 0.92; 95% CI, 0.82-1.03).

## Discussion

In this propensity-matched analysis of patients with stage IV, nonsquamous, non–small cell lung cancer, we found that first-line use of carboplatin-pemetrexed compared with carboplatin-paclitaxel was associated with a lower risk of hospitalization and improved survival. We reported a 5.3% reduction in the 30-day hospitalization risk among patients receiving carboplatin-pemetrexed, corresponding to a 20% reduction in hospitalizations in the 30 days following chemotherapy initiation. Treatment with carboplatin-pemetrexed was also associated with significant improvement in the median survival, by 1.4 months (*P* = .005). These findings are notable because carboplatin-pemetrexed and carboplatin-paclitaxel are the 2 most commonly used cytotoxic chemotherapy regimens for advanced lung cancer, and, to our knowledge, there are no large prospective studies directly comparing these regimens. Our main analysis includes 2182 patients with nonsquamous non–small cell lung cancer with a median age of 73 years, whereas existing prospective studies of each of these regimens are skewed toward younger, more pathologically heterogeneous patient populations.^20^

Although the first-line treatment of advanced non–small cell lung cancer is in rapid evolution today,^21,22^ cytotoxic chemotherapy remains an important part of the lung cancer treatment arsenal. Pembrolizumab monotherapy is now a guideline-recommended first-line therapy for patients with programmed cell death ligand 1 (PD-L1)–positive metastatic lung cancer; however, more than half of patients in this relatively favorable subgroup experience progression within a year or less.^21^ Platinum-based doublets are the recommended second-line therapy after progression following first-line immunotherapy. For patients with PD-L1–negative lung cancer, combination chemoimmunotherapy with a platinum-based doublet is an emerging standard of care.^22^ In either setting, platinum-based doublet chemotherapy will continue to play an important role for most patients with metastatic lung cancer, highlighting the ongoing relevance of the findings reported here.

To place our findings in context, it is helpful to review how carboplatin-pemetrexed and carboplatin-paclitaxel became the predominant cytotoxic chemotherapy regimens for treatment of metastatic, nonsquamous, non–small cell lung cancer. In 2002, a study from the Eastern Cooperative Oncology Group^23^ showed apparent equivalence of 4 chemotherapy doublets in the treatment of advanced non–small cell lung cancer, with a median survival of approximately 8 months for all regimens. Carboplatin-paclitaxel was the only noncisplatin combination evaluated, and subsequent widespread adoption of carboplatin-paclitaxel was based in part on its favorable toxicity profile. Pemetrexed was not approved for treatment of lung cancer until 2008, when Scagliotti and colleagues reported that the combination of cisplatin and pemetrexed appeared to offer improved survival in comparison with cisplatin and gemcitabine, although only for the nonsquamous histologic subgroup.^24,25^ Cisplatin remains infrequently used for Medicare patients^26^ owing to toxicity and patient comorbidity, and a subsequent study^27^ supported the use of pemetrexed with carboplatin instead of cisplatin. In this way, carboplatin-paclitaxel and carboplatin-pemetrexed became the 2 preferred chemotherapy regimens for nonsquamous non–small cell lung cancer without having been directly compared with each other in a large prospective study. The PointBreak study^28^ (n = 939) compared these regimens in the context of universal bevacizumab use, and the PRONOUNCE trial^29^ (n = 361) compared carboplatin-pemetrexed with carboplatin-paclitaxel-bevacizumab, with both studies reporting null results for their primary outcomes. In addition, the various maintenance strategies within these studies and the differences in the use of bevacizumab have left a number of unresolved questions about the comparative efficacy of these regimens.

A key objective of the present study was to investigate new outcome measures of acute hospitalization applicable to patients receiving chemotherapy. We chose 30-day hospitalization risk as the primary outcome because the period immediately after chemotherapy initiation is known to be high risk for hospital admission and because short-term outcome measures are less likely to be affected by the competing risk of mortality. Our study confirms a startlingly high 30-day hospitalization risk among patients with lung cancer initiating chemotherapy (ie, 20.7% to 26.0% in the propensity-matched cohorts). Another measure evaluated here, the 90-day mean hospital-free survival time, provides insight into the competing risks of hospitalization and death. We contend that these measures, readily extractable from claims data, can serve as valuable proxies for adverse events during chemotherapy. A third hospitalization measure, 90-day cumulative hospitalizations, showed no difference between patients receiving either of the 2 chemotherapy regimens. This is likely because improved survival among patients receiving carboplatin-pemetrexed translated into greater time at risk of hospitalization, leading to a similar number of cumulative hospitalizations with improved hospital-free survival time.

Overall, we found that carboplatin-pemetrexed therapy was associated with a lower risk of hospitalization and superior survival outcomes compared with carboplatin-paclitaxel therapy—a clear win for carboplatin-pemetrexed therapy from the effectiveness perspective. The relative economic value of these treatments is less clear-cut. Using the average sales price from the fourth quarter of 2017, we found that the drug cost is approximately $6000 for a 3-week cycle of carboplatin-pemetrexed and less than $50 for a 3-week cycle of carboplatin-paclitaxel. Use of bevacizumab at standard doses adds more than $8000 per treatment cycle with either regimen.^30^ We did not perform a formal cost-effectiveness analysis; however, any anticipated reduction in hospitalizations is very unlikely to offset the incremental cost of substituting pemetrexed for paclitaxel. Still, patients may derive substantial quality-of-life benefit from avoiding hospitalization.

We did not find an association of bevacizumab use with either hospitalization or survival. The addition of bevacizumab to carboplatin-paclitaxel therapy led to a 2-month survival improvement in a prospective clinical trial,^31^ and retrospective analyses have confirmed improved survival with bevacizumab use.^32,33^ However, our finding of no survival advantage with the addition of bevacizumab to chemotherapy is consistent with observational studies that have challenged the utility of bevacizumab for elderly patients.^33,34^ As immunotherapies become first-line therapies for many patients with advanced non–small cell lung cancer, it may be appropriate to abandon costly and underwhelming vascular endothelial growth factor inhibitor therapies for most elderly patients with lung cancer.

### Strengths and Limitations

The strengths of our analysis included the use of the linked SEER-Medicare data, which are richly annotated with cancer diagnosis information, including month of diagnosis, cancer stage, and histologic findings. Comprehensive eligibility and claims data provided high confidence that outcomes assessments were comprehensive, and the use of a propensity-matched analytic design provided strong support for the assumption of comparable patient groups. A key limitation of our analysis was that we could not fully adjust the analysis for the use of bevacizumab, an active cancer therapy that was imbalanced across the patient groups. However, our exploratory analysis provided reassurance that bevacizumab use did not appear to be an important determinant of either hospitalization or survival outcomes. Also, we could not exclude the possibility that unmeasured factors were associated with both the choice of chemotherapy regimen and the risk of hospitalization.

## Conclusions

Treatment with carboplatin-pemetrexed for elderly patients with advanced lung cancer was associated with a 5% absolute reduction in 30-day hospitalization risk compared with that for treatment with carboplatin-paclitaxel. Carboplatin-pemetrexed therapy was also associated with more favorable survival. As immunotherapy supplements and replaces cytotoxic chemotherapy in the first-line treatment of metastatic non–small cell lung cancer, these results remain relevant to first- and second-line treatment selection and are useful for benchmarking important treatment outcomes in the Medicare population.

## Notes

Supplement 1. **eTable.** Hospitalization and Survival Outcomes in Unmatched Patients Receiving Carboplatin-Based Chemotherapy With Paclitaxel or Pemetrexed

Supplement 2. **Data Sharing Statement**
