# Supplementary material for: Hospitalization and Survival of Medicare Patients Treated With Carboplatin Plus Paclitaxel or Pemetrexed for Metastatic, Nonsquamous, Non–Small Cell Lung Cancer
Source: JAMA Netw Open. 2018 Oct 5;1(6):e183023. doi: 10.1001/jamanetworkopen.2018.3023 (PMC6324452; doi:10.1001/jamanetworkopen.2018.3023)
Supplement: Supplement 1. — eTable. Hospitalization and Survival Outcomes in Unmatched Patients Receiving Carboplatin-Based Chemotherapy With Paclitaxel or Pemetrexed [file jamanetwopen-1-e183023-s001.pdf]

Supplementary Online Content

Brooks GA, Austin AM, Uno H, Dragnev KH, Tosteson ANA, Schrag D. Hospitalization and survival of Medicare patients treated with carboplatin plus paclitaxel or pemetrexed for metastatic, nonsquamous, non–small cell lung cancer. *JAMA Netw Open*. 2018;1(6):e183023. doi:10.1001/jamanetworkopen.2018.3023

eTable. Hospitalization and Survival Outcomes in Unmatched Patients Receiving Carboplatin-Based Chemotherapy With Paclitaxel or Pemetrexed

This supplementary material has been provided by the authors to give readers additional information about their work.

© 2018 Brooks GA et al. *JAMA Network Open*.

eTable. Hospitalization and Survival Outcomes in Unmatched Patients Receiving Carboplatin-Based Chemotherapy With Paclitaxel or Pemetrexed

**Outcome**	**Carboplatin-paclitaxel** (n = 1487)	**Carboplatin-pemetrexed** (n = 1823)	**Difference**	**p-value**
30-day hospitalization risk	25.9% (23.7, 28.1)	21.3% (19.5, 23.2)	-4.6% (-7.5, -1.6)	0.002
90-day hospitalization count, mean	0.59 (0.55, 0.63)	0.53 (0.50, 0.57)	-0.06 (-0.11, 0.00)	0.06
90-day mean hospital-free survival time, days	78.3 (77.1, 79.6)	82.1 (81.2, 83.0)	3.8 (2.2, 5.3)	<0.001
90-day overall survival probability	75.4% (73.2, 77.6)	81.8% (80.0, 83.6)	6.4% (3.6, 9.2)	<0.001
Median overall survival	7.8 months (7.1, 8.5)	8.9 months (8.4, 9.4)	1.1 months (0.2, 2.0)	0.02

Parentheses show 95% confidence intervals.

© 2018 Brooks GA et al. *JAMA Network Open*.
