# Supplementary material for: Hospitalization and Survival of Medicare Patients Treated With Carboplatin Plus Paclitaxel or Pemetrexed for Metastatic, Nonsquamous, Non–Small Cell Lung Cancer
Source: JAMA Netw Open. 2018 Oct 5;1(6):e183023. doi: 10.1001/jamanetworkopen.2018.3023 (PMC6324452; doi:10.1001/jamanetworkopen.2018.3023)
Supplement: Supplement 2. — Data Sharing Statement [file jamanetwopen-1-e183023-s002.pdf]

Data Sharing Statement

Data

Data available: No

Additional Information

Explanation for why data not available: Our analysis uses a publicly-available data linkage (the SEER-Medicare linked data) that is curated by the National Cancer Institute. Our data use agreement prohibits us from sharing these data ourselves.
